# Optical Navigation Sensor for Runway Relative Positioning of Aircraft during Final Approach

**DOI:** 10.3390/s21062203

**Published:** 2021-03-21

**Authors:** Antal Hiba, Attila Gáti, Augustin Manecy

**Affiliations:** 1 Institute for Computer Science and Control (SZTAKI), Eötvös Loránd Research Network (ELKH), H-1111 Budapest, Hungary; matgati@sztaki.hu; 2 ONERA—The French Aerospace Laboratory, 31000 Toulouse, France; Augustin.Manecy@onera.fr

**Keywords:** runway detection, on-board vision system, real-time image processing, automatic landing

## Abstract

Precise navigation is often performed by sensor fusion of different sensors. Among these sensors, optical sensors use image features to obtain the position and attitude of the camera. Runway relative navigation during final approach is a special case where robust and continuous detection of the runway is required. This paper presents a robust threshold marker detection method for monocular cameras and introduces an on-board real-time implementation with flight test results. Results with narrow and wide field-of-view optics are compared. The image processing approach is also evaluated on image data captured by a different on-board system. The pure optical approach of this paper increases sensor redundancy because it does not require input from an inertial sensor as most of the robust runway detectors.

## 1. Introduction

The importance of safety is indisputable for any aircraft from unmanned aerial vehicles (UAVs) to commercial flights. Sensor redundancy is the basic element for safety critical systems. There are multiple types of redundancy from sophisticated voting methods [1] to analytical redundancy [2,3]. In sensor fusion, different types of sensors measure the same phenomenon to achieve more robustness. Beyond fault detection capability of multiple sensors, sensor fusion can give improved performance compared to each component [4,5,6].

For fixed-wing UAVs and aircraft, the most dangerous flight phases are final approach and landing [7] where precise navigation data are the key factor. Navigation data are acquired from Global Navigation Satellite System (GNSS) and Instrument Landing System (ILS) if ILS is available at the target landing site. Numerous extensions and additional sensors were introduced to aid piloted landing and support autonomous landing. Charnley [8] presented an auto-landing system in 1959 based on ILS and barometer for the first part of the approach (above 30.5 m height) and radio altimeter and magnetic leader cable in horizontal plane for the landing part. Today, augmented GPS solutions can also meet the requirements of the CAT IIIb approach [9]. The increasing demand for aerial mobility and transport motivates researchers to develop navigation solutions with less infrastructural dependence (radio beacons and satellites). Camera-based navigation sensors provide more autonomy in the case of unavailable or degraded GNSS, and enhance robustness in sensor fusion. Camera sensors are completely passive and non-cooperative which is beneficial for military use, and the lack of signal emission is also important in future civil utilization where dense population of UAVs could interfere and cause signal pollution.

Vision-based navigation in general has three main components: sensor type (ultraviolet [10], infrared [11,12], visible RGB/mono [11,13], stereo [14]), a priori data (terrain model [10], satellite images [15], UAV images [16], 3D position of key features [11,13,14]), and data accumulation (single frame [11,13,14] and optic flow/SLAM [6,16,17,18,19,20]). Runway relative navigation for autonomous landing is a special case. The runway and its standardized markings are useful key features with known 3D positions, and they can be applied in the most critical phase of the approach when the aircraft has low altitude and thus highest precision is required. All-weather marking solutions exist (infrared (IR) and ultraviolet (UV)), however detection of current runway markers requires good visibility conditions. The vehicle has high speed and long-range detection is required. Vision-based solutions for autonomous landing of fixed-wing aircraft always use information about the target runway and the detection of relevant runway features is the starting point of navigation data extraction.

If a 3D runway model is available, one can generate an artificial image of it from an estimated 6D pose (3D position and 3D attitude), then it can be fine tuned based on detection in the real camera image. In [10], a GPS-IMU (Inertial Measurement Unit) 6D pose is used to generate a synthetic image of the runway ultraviolet lights, and it is registered with the actual detection in the UV image of the runway. UV is beneficial, because almost no natural background radiation is detectable on the surface of the Earth below 0.285 micron. Binary images of a complete polygon model of the runway with taxiway exits is generated in [21] from a neighborhood of the IMU/GPS pose, and these images are compared with the shifted hue channel of the HSV version of an RGB camera image to get the best fit. From IMU pose and runway geoinformation, four edges are rendered in [12] and line features are fitted in the real image which defined the homography from the synthetic image to the real image. Four corner points of the runway (parallel lines with four points) are also enough for 6D pose calculations. Hecker et al. [11] use the Aeronautical Information Publication database to get 3D runway corner points, and this model is projected to camera image using global orientation derived from IMU and estimated position of the camera. The projection is fine-tuned in a region of interest (ROI) based on line contour features. Hecker et al. also use infrared camera along with RGB camera, and detection is performed on both sensors. The IR sensor was able to detect the runway surface from further distance (2 km), while runway markers were only visible in RGB images (<600 m). The same team introduces integrity check of a GNSS/INS based positioning and tracking system augmented by an optical navigation sensor [22].

Visual-inertial odometry (VIO) [23,24] and visual-inertial simultaneous localization and mapping (VI-SLAM) and their extensions with GNSS [17,18] is the current development trend in visual navigation, but we still need better vision sensors in these complete multisensor solutions. Vision senors in navigation are used for enhancement of IMU/GPS Kalman filter solutions; however, most of the runway relative navigation methods rely on the estimate coming from the IMU/GPS which dependence can be eliminated with robust image processing.

Robust detection of a runway from long distance (>1 km) without a good initial guess is challenging. Deep learning solutions exist for runway detection in remote sensing images [25]. Current long-range solutions for aircraft imagery utilize at least IMU attitude information to obtain ROI for the runway detection. Within 1 km from a 30 m width runway it is possible to define vision-only detection without any IMU support. The detection of runway features is also necessary for ROI-based methods, thus runway detection in camera images has to be addressed for any navigation sensor which is dedicated for the final approach. Obstacle detection during landing also requires runway detection [26,27,28] to identify the target area.

Runway detection should be done in IR and RGB/monochrome camera images. IR has the advantage of weather independence, however, it has lower resolution, higher price, and lack of texture details. IR is beneficial for all-weather complete runway detection from longer distances, while RGB can provide detailed information about threshold marker and center line at the end of the approach and during landing and taxiing [11]. Line features are straight edges which can be detected with horizontal/vertical Sobel filters, which are separable 2D filters thus they can be applied to the whole image at low computation cost. Hough transform is used in [13,29,30,31], which gives a complete representation of possible lines, but has higher computational cost and short segments on the same line gives as high response as a long continuous line and the separation of lines with same orientation and close distance is hard. Detection of short line features is not possible with a complete Hough transform. Specialized line segment detectors LSD [32] and EDLines [33] are used in [34]. In [35], the authors present a family of edge detectors for various orientations. Beyond line feature detection, the registration of a synthetic image with known 6D pose [36,37] and registration of real images from database [38] are also applied to solve the runway detection problem.

Most of the runway detection methods are developed and tested in a simulator environment where the runway area has perfect contours and markings. In real life, the side line and threshold line of the runway is often missing or its width is too narrow for detection from longer distances, thus complete runway detectors need to rely on texture difference or contours in special color channels to detect the tarmac area. The difference between the tarmac and the environment has much higher variability (seasons and different runways) than the difference between the tarmac and the threshold marker. Usage of an image database for matching has high computational complexity and fine tuning is still necessary to have precise detection results. All existing methods focus on the complete runway detection with the assumption of flat runways. This assumption is not true for km long targets; furthermore, many airfields have a bump at the middle to support acceleration and deceleration of aircraft. Threshold markers (white bars) can support relative navigation without any other features they are designed for good visibility and they lay in a planar area. The pattern of these white bars provide additional information for filtering out false detections, and makes it possible to detect runway in full frames without initial guess coming from IMU/GPS measurements. Our detection method is designed for the last phase of the approach (600–50 m from threshold of 30 m width runway), where the most precise navigation data is required. This paper does not cover the landing and taxiing phase, where radio altimeter, center line, and side line detection (they are in close proximity during landing phase) can be used.

Theory and technology for vision-based runway relative navigation were ready for real flight validation in 2018. One major goal of the H2020 VISION (Validation of Integrated Safety-enhanced Intelligent flight cONtrol) project was to build a fixed-wing experimental UAV with a cutting edge stereo sensor [14] and pair of monocular sensors with different field of view (FOV) to validate vision-based sensor performance in real flight situations and use visual sensors for integrity monitoring and sensor fusion [5,24].

Optical navigation sensors have great potential in sensor fusion setups. Different sensors have different bias and standard deviation with low probability for having fault at the same time. Avionics of fixed-wing UAVs and manned aircraft have IMU for attitude, GNSS for position, Satellite-based Augmentation System (SBAS) for the reduction of GNSS bias, radar altimeter for landing support, barometric altimeter, airspeed sensor, and compass for yaw angle measurement. The bias and standard deviation of optical sensors are theoretically decreasing during the approach, which attracts interest of researchers and industry to add them to the sensor setup. Camera sensors are relatively cheap and have low power/weight footprint; however, on-board image processing requires a payload computer, and image processing adds >20 ms delay between the measurement (exposure) and the navigation data, which makes the integration of optical sensor data hard into the sensor fusion filter.

This paper presents the methods and real flight test results for the pair of monocular RGB sensors. We focus on raw image-based navigation performance, because we consider the vision sensor as a building block for integrity test and sensor fusion with IMU/GNSS. Integration of IMU units into the camera can be beneficial for robotic applications; however, the methods which are described here do not need IMU. The core of our runway detection is a robust threshold marker detection which can support IMU-free runway detection when the marker becomes visible and also provides superior precision at the end of the approach. The sensor hardware is also described with the achieved results of the on-board real-time operation during flight tests. The results of different FOV sensors are compared. Beyond the own flight test results, the image processing method is also tested on the real flight data which was provided by the C2Land project [11]. The paper summarizes the main concepts for navigation data extraction from image features of a runway (Section 2), presents the image processing method for threshold marker detection (Section 3), introduces the experimental platform and the monocular image sensor hardware (Section 4) and finally discusses the raw image-based navigation results (Section 5).

## 2. Navigation Data from Runway Features

**Figure 1:**
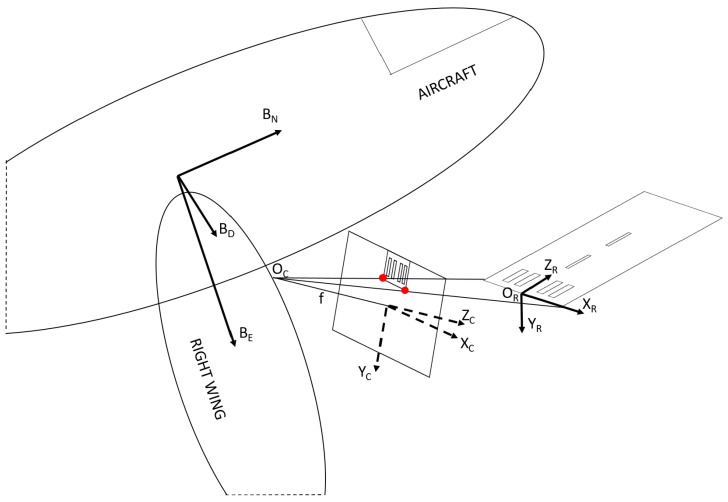
Relevant coordinate systems of vision-based navigation sensor. The origin of runway coordinate system $R_{XYZ}$ is known in GPS LLA system with the 3D heading angle of the runway. The transformation between $R_{XYZ}$ and camera coordinate system $C_{XYZ}$ is the question, while transformation between aircraft body system $B_{NED}$ and the camera $C_{XYZ}$ is determined by calibration.

Navigation always defines coordinates according to a reference coordinate system. The most common global reference is the Latitude Longitude Altitude (LLA) of the WGS’84 system, which defines a point on the geoid surface model of the Earth and altitude above that point. For low-range aerial vehicles, the most common navigation frame is the North East Down (NED) Cartesian coordinate system which is defined by an origin on the surface, and flat earth approximation gives the North and East unit vectors. Figure 1 presents the relevant coordinate systems and their relations in 3D. Runway features are detected in the 2D image of the camera which is on a plane in the camera frame at $Z_{C}=f$, where *f* is the focal length of the pinhole camera. From these feature points in the image and physical position coordinates of these features in 3D, one can get the 6D relative pose (3D position and 3D attitude) to any global Cartesian systems (global NED, $R_{XYZ}$). The relation of the camera $C_{XYZ}$ and the aircraft body system $B_{NED}$ can be obtained through camera calibration.

In the most general case, 3D positions of features are given with their 2D projections into the image (n≥4). The question of Perspective-n-Point (PnP) is to find 6D pose of a calibrated camera [39].

(1) $$s\cdot \left[\begin{array}{c} u\\ v\\ 1 \end{array}\right]=\left[\begin{array}{ccc} f_{x} & \gamma & u_{0}\\ 0 & f_{y} & v_{0}\\ 0 & 0 & 1 \end{array}\right]\left[\begin{array}{cccc} r_{11} & r_{12} & r_{13} & t_{1}\\ r_{21} & r_{22} & r_{23} & t_{2}\\ r_{31} & r_{32} & r_{33} & t_{3} \end{array}\right]\left[\begin{array}{c} x\\ y\\ z\\ 1 \end{array}\right]$$

Equation (1) describes the homogeneous transformation of the projection from homogeneous physical coordinates ${\left[x,y,z,1\right]}^{T}$ to homogeneous image coordinates $s\cdot {\left[u,v,1\right]}^{T}$, where *s* is a scaling factor because many physical points have the same image projection. The first matrix of the right side describes the intrinsic parameters of the pinhole camera, which are known from calibration. Focal length in pixels $f_{x},f_{y}$ are derived from the physical dimensions of a sensor pixel, skew γ (zero for most optics), and image coordinates of the principal point $u_{0},v_{0}$ (the center of the image for a high quality optics). The second matrix encodes the extrinsic parameters which are the 3D translation ($t_{1}$, $t_{2}$, $t_{3}$) and 3D rotation (3 × 3 matrix $r_{11}$ to $r_{33}$) of the camera. PnP defines the unknown parameters of the extrinsic matrix, which encodes the 6D pose of the camera.

Runway features lie on a surface in 3D thus more specific pose estimation methods are available. Homography defines transformation between 3D positions on a surface (z=0) to 2D projections in a camera image. In Equation (1), the column of the extrinsic matrix ($r_{13}$, $r_{23}$, $r_{33}$) which corresponds to *z* can be excluded.

(2) $$s\cdot \left[\begin{array}{c} u\\ v\\ 1 \end{array}\right]=\left[\begin{array}{ccc} f_{x} & \gamma & u_{0}\\ 0 & f_{y} & v_{0}\\ 0 & 0 & 1 \end{array}\right]\left[\begin{array}{ccc} r_{11} & r_{12} & t_{1}\\ r_{21} & r_{22} & t_{2}\\ r_{31} & r_{32} & t_{3} \end{array}\right]\left[\begin{array}{c} x\\ y\\ 1 \end{array}\right]$$

Equation (2) describes this special case were the 3 × 3 matrix which describes the overall transformation is called the homography matrix and 6D pose of the camera can be decomposed from it [40]. Features on the same plane surface can also support camera motion estimation and calibration [41,42].

In the VISION project [43] beyond homography, the 3-point algorithm of Li et al. [44] was also used for runway relative navigation, because it has minimum number of image features which can be tightly coupled into the sensor fusion. The three points are the right and left corner points of the runway and the vanishing point of the side lines. *D* denotes the known width of the runway, and $r_{R}$ and $r_{L}$ are the 3D image vectors with real metric sizes pointing from the camera frame origin to the detected corner features on the image surface (z=f). $q_{R}=\lambda_{R}\cdot r_{R}$ and $q_{L}=\lambda_{L}\cdot r_{L}$ are the vectors pointing from the camera frame origin to the physical corners. Let $t=q_{R}-q_{L}$ be the physical vector of the runway threshold and $r_{V}$ be the image vector of the vanishing point. $\lambda_{R}$, $\lambda_{L}$ depths are the two unknowns of Equation (3). *t* and $r_{V}$ should be perpendicular, while the length of *t* is equal to the width of the runway (*D*).

(3) $$\left\{\begin{array}{c} t\cdot r_{V}=0\\ t\cdot t=D^{2} \end{array}\right.$$

The camera frame to runway frame rotation matrix R can be defined by the normalized (hat) version of the perpendicular *t* and $r_{V}$ vectors (Equation (4))

(4) $$R=\left[\begin{array}{c} \hat{t}\\ \hat{r_{V}}\times \hat{t}\\ \hat{r_{V}} \end{array}\right]$$

$T_{c}=\left(q_{L}+q_{R}\right)/2$ is the vector in camera frame pointing to the runway frame origin. Translation of the camera in runway frame is $T=-R\cdot T_{c}$. If the navigation problem is solved by homography, we just calculate the three image features which corresponds to the result; however, the above equations give insight into the close connection of these features to the runway relative navigation data.

## 3. Runway Marker Detection

Runway detection defines achievable accuracy of later steps in sensor fusion [24] or visual servoing [45,46]. Runway markers, especially the threshold bars, are designed for good visibility and provide relative visual navigation for the pilots. The number of bars indicates the width of the runway and these large long rectangles give enough features for homography. We assume that the real size and relative position of threshold bars are known, and they are on a planar surface (homography). We use the runway relative metric coordinate system with origin at the center of the bottom line of the threshold bars (Figure 1).

**Figure 2:**
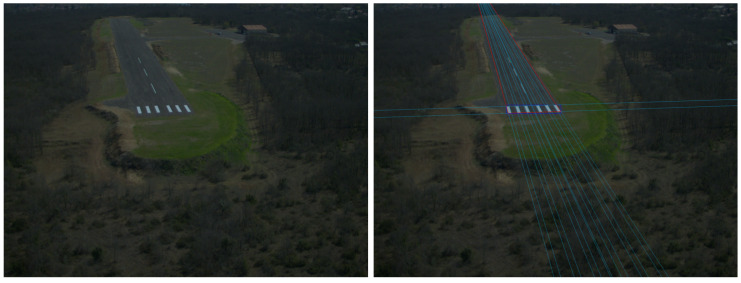
Original RGB image from an approach to Septfonds runway (**left**) and the output of the runway marker detection presented in the undistorted image (**right**).

Threshold bars are transformed rectangles in the captured images. We assume that spatial order and distance/width ratio ($R_{WD}$) of consecutive rectangles is similar to the top-view template (representation). It is true for possible approach trajectories towards the pattern from $-Z_{R}$ direction, in general, only the cross-ratio can be used which describes relation of four collinear points. The detection has two main stages: detection of possible transformed rectangles and then representation matching of the threshold marker. The representation consists of 4–16 rectangles with given distance/width ratios. At Septfonds airfield we have 2 × 3 bars, the width of a bar is 1.5 m and the distance between the the two bars in the middle is 6 m while the other bars have 4.5 m separation. An example image and the corresponding detection are presented in Figure 2. Optics can have radial distortion; however, in the case of small distortion, the detection of short line features is possible without undistortion of the image. Undistortion is applied after the runway marker representation matching inside the ROI of the detected threshold bar area.

### 3.1. Detection of Threshold Bar Candidates

**Figure 3:**
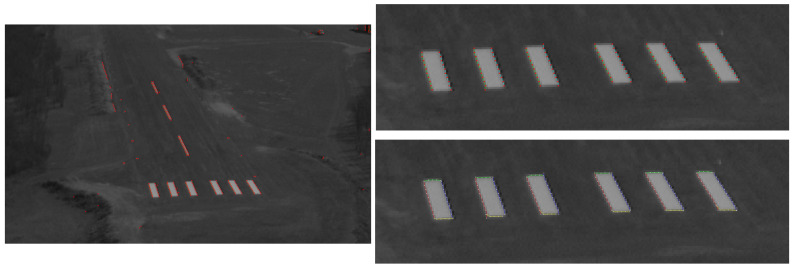
Runway area of the grayscale image, all steps done on the whole image. Vertical edges (**left**) and line chains to quadrangles (**right**). Horizontal edges and chains are also computed.

Our approach focuses on the threshold bars which are brighter quadrangles on the runway surface. The image is converted to grayscale, and the transposed image is also created to calculate horizontal and vertical edge features in parallel. For vertical edge detection, a 3 × 3 Sobel filter is used on each 2nd row of the image and on its transposed version (horizontal edges). Figure 3 presents the result of the Sobel calculations for the vertical edges with both bright-to-dark (right) and dark-to-bright (left) edges where the local extreme values are selected above an absolute value threshold. After filtering out singletons, we start to build right and left edge chains (similarly in the transposed image for bottom and top edge chains). Edge chains are trees in general, however they are almost straight lines for the threshold bars (small branches or top/bottom corner area added in the case of large roll angles). Pruning of shorter branches results right and left line segments for the threshold bars. Pairing of right and left segments is done through the horizontal segments which should intersect both a right and left segment to create a clamp. Clamps can have more than two intersecting horizontal segments thus we need to choose the two extremal one which leads Left-Right-Bottom-Top (LRBT) complete quadrangles and also LRT and LRB detections which also define quadrangles. We do not care much about other false detected clamps, because we apply representation match for the threshold marker to choose the clamps (transformed rectangles) which represent the bars of the threshold marker.

### 3.2. Representation Matching of the Threshold Marker

Detection of bright quadrangles is only sufficient inside a ROI which is often given by an IMU-based guess about the position of the runway in the image. For an independent full-frame detection, we need to assign quadrangle candidates to positions in a complex pattern (representation). In the Septfonds case, we need to assign 6 bar indices to 6 quadrangles from possibly a hundred of candidates.

The general idea of representation matching is described in [47]. We derived a special case with some additional heuristics to meet our operational needs. A representation can have elements of multiple types (representation of a face has eyes, ears, hair, etc.) and each has a cost of goodness. The key is the cost of relative pose of the elements in the image compared to an ideal representation were each element can be connected to multiple elements through the cost functions. We have a simple representation with only bars, and for each bar we have relative pose cost only for the consecutive bar. At this point, *M* transformed rectangles are given in the image and the threshold marker (*B* bars) is required (if it presents in the image).

**Figure 4:**
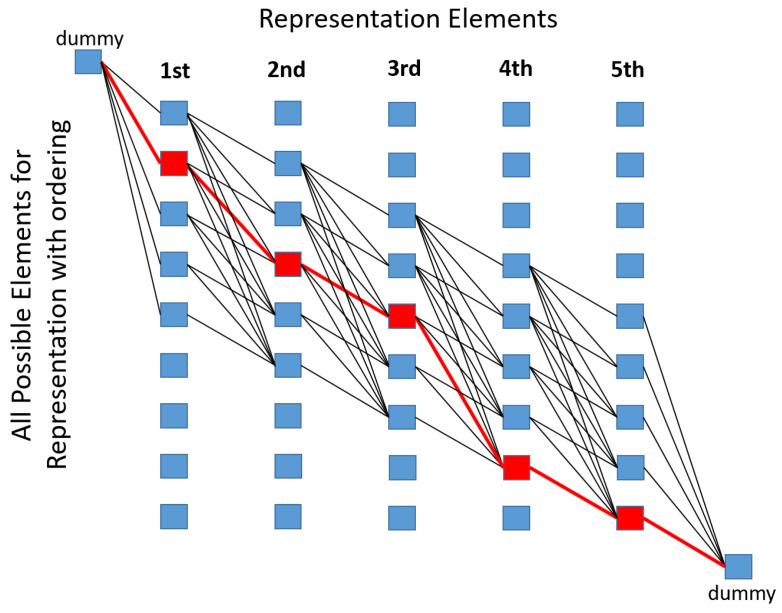
Simplest case of dynamic programming table (M=9,B=5). Nodes are rectangles at a given position in the representation of the threshold marker. Without ordering, two consecutive columns should be fully connected (except same row). Edges represent cost of a given succession in the pattern (goodness of relative fit). Red line is the minimum cost route between the two external dummy node, and it gives the optimal fit.

We assume that the spatial order of rectangles in the image is the same as in the ideal threshold bar pattern (*B* rectangles as a top-view pattern). The bar candidates can be ordered according to their x coordinate, and it is sure that any rectangle in a fit cannot be followed by a rectangle with lower index. To evade exhaustive search of all possible combinations, dynamic programming is applied. Figure 4 shows the simplest case of the dynamic programming table with ordered elements. Each edge in the table represents a succession cost in the pattern, and each route between the two dummy nodes corresponds to a pattern fit (selected candidates for each position in the pattern). In our model, only consecutive elements of the pattern interacts in the cost function which yields *M* rows and *B* columns in the table. However, we let missing elements which requires additional edges between elements of nonadjacent columns thus the number of edges is multiplied by 2. Spatial ordering of bar candidates also makes it possible to further decrease the edges to be calculated in the table by excluding far elements in successor search. This excursion destroys theoretical optimality, however, we have already forced to use heuristic approach to define the cost functions.

Each edge in Figure 4 represents a succession cost assuming that the two rectangle candidates are chosen for the two consecutive positions in the representation. We have the following assumptions which are mainly true in the case of an approach towards the runway marker:

- Line segments of the threshold bars are not longer than the sides of the quadrangle: number of pixels beyond corners.
- The two TB and two LR segments have nearly the same length: $\frac{difference^{2}}{length^{2}}$.
- Consecutive candidates have nearly same width and height: $\frac{difference^{2}}{length^{2}}$.
- Vertical directions for consecutive candidates nearly the same: $\frac{1}{cos^{2}\left(diff\right)}-1$.
- The ratio $R_{WD}$ of width and distance between pattern elements is the same as in the top-view pattern: $R_{WD}-\frac{distance}{width}$.

**Figure 5:**
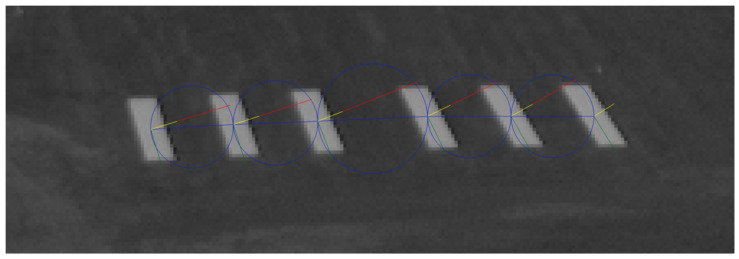
Visualization of the components of succession cost. Center points of candidates are connected by blue lines, and a circle represents the points with equal distance from both center points. Yellow line is the normal vector of the main (longitudinal) orientation of a bar and the red vector has $R_{WD}*width$ length.

The first two elements inhibit the false bar detections while the other components lead the optimization towards the correct identification of the bars inside the threshold marker. The weighted sum of the cost components is used with additional penalty on a missing element in the representation match. Weights are not sensitive parameters, they just normalize the components. Figure 5 visualizes the succession costs.

Dummy nodes are connected to all possible first and last candidates with zero cost edges. In the example of Figure 4, we have 5 representation elements (missing elements are not allowed) in the representation, thus the last 4 candidates cannot be the first element, similarly the first 4 candidates cannot be chosen as the last element. The candidates are ordered, which means that only candidates with higher indices are available as a successor. Succession costs (last 3 entries of the cost function list) are weights on the edges between the candidates, while candidates also have their own goodness (first two entries of the cost function list). The solution is the minimal cost route between the two dummy nodes which can be obtained by the Dijkstra algorithm.

At this point, we have the best possible matching of quadrangle candidates for the threshold marker. The cost function makes it possible to drop weak detections (typically when the marker is not present in the image) which also increases the robustness. The threshold marker area is further processed to obtain better accuracy.

### 3.3. Fine Tune and Output Generation

**Figure 6:**
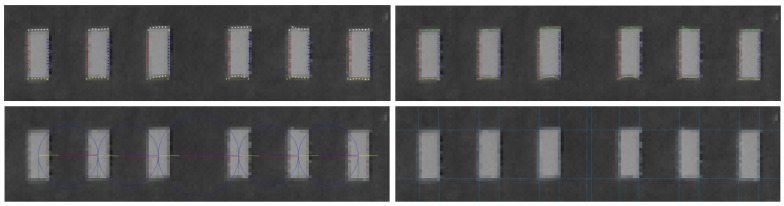
Bar detection is fine-tuned on the top-view projection of the detected marker.

After runway marker detection, we can use the homography which is defined by the corner features of the bars and their known 3D positions to create a top-view from the threshold marker area. Figure 6 shows the top-view on which we repeat the complete detection algorithm without downsampling but with undistortion. The result is the final detection of threshold bars and the corresponding homography.

The output of the image processing is the runway relative 6D pose and the three key image feature points which are equivalent representation of the 6D pose. Key image feature points are used in tight coupling into the navigation/control. Representation match is useful for any special land marking detection where the elements of the pattern can be detected with high recall.

## 4. On-Board Vision System

**Figure 7:**
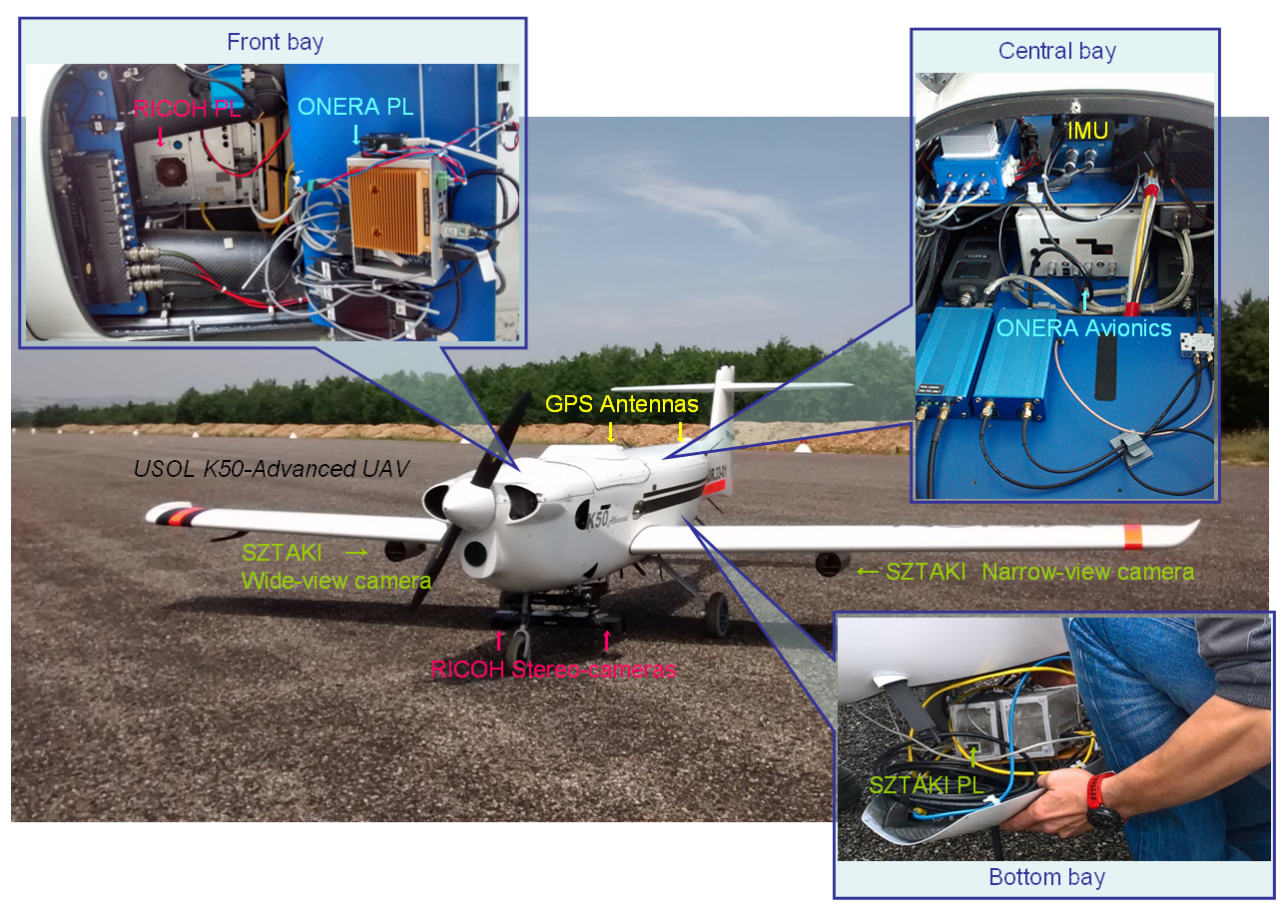
USOL (Spanish SME) K50 UAV experimental platform (60-kg fixed-wing UAV of 4 m wing span) with complete sensor and on-board computer setup of the H2020 VISION project [43].

Our vision-based navigation sensor which is described in this paper (SZTAKI sensor) was a building block of a complex UAV system (Figure 7). It is designed for real flight validation of navigation and guidance performance recovery from sensor failures (e.g., degradation of GPS/SBAS or ILS) [5]. The experimental platform was equipped with an avionic (computer and navigation sensors: 9-axis IMU, GNSS, barometer, and pitot tube) developed by ONERA, a stereo-vision sensor of RICOH company, and SZTAKI sensor based on 2 different field-of-view (FOV) cameras. RICOH stereo and SZTAKI monocular sensors are devoted to runway relative navigation. Other navigation sensors (IMU, GNSS, and barometer) are used for ground truth collection. On-board emulated ILS and GNSS degradation models are applied to emulate failures and degradations in the guidance performance recovery tests where the independent optical sensors provided the necessary additional information. Stereo sensor was also used for obstacle detection on the runway. All the payloads were time-synchronized by the pulse per second (PPS) signal of the central GPS receiver. Information of vision-based sensors were processed in real-time and collected by ONERA payload for ground-station feedback and for the navigation filter [14].

The SZTAKI sensor is developed by our team and dedicated to monocular vision-based navigation. The system has two GEthernet cameras (2048 × 1536 image resolution, 7.065 × 5.299 mm physical sensor size) with narrow angle (12 mm focal length, 32.8· FOV) and wide angle (6 mm focal length, 60.97· FOV) optics. Both cameras can see the runway during the approach which makes the comparison of the different FOV sensor outputs possible. This setup is good for scientific experiments. In a real use case, the FOVs can be narrower, because if the narrow FOV sensor cannot see the runway, the wide FOV system surely sees it during final approach assuming small/medium deviance in the position/orientation.

**Figure 8:**
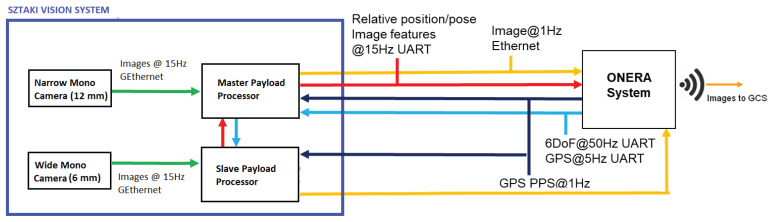
Components and interfaces of the monocular vision sensor with two cameras and two payload computers.

Figure 8 presents the components and communication interfaces of the monocular navigation sensor. Each camera has a dedicated Nvidia TX1-based image processing computer (256 CUDA Cores, Quad-core ARM Cortex-A57, 4 GB LPDDR4). One of the computers plays a master role and only this subsystem provides the output of vision system.

All calculations are done on-board, while the ONERA payload sends diagnostics with downsampled camera images and state variables through wifi to the ground station. Payload computers must have small power/weight footprint and high computational capacity. RS232 communication interfaces are used for state information exchange and UDP for transmission of the diagnostic images.

**Figure 9:**
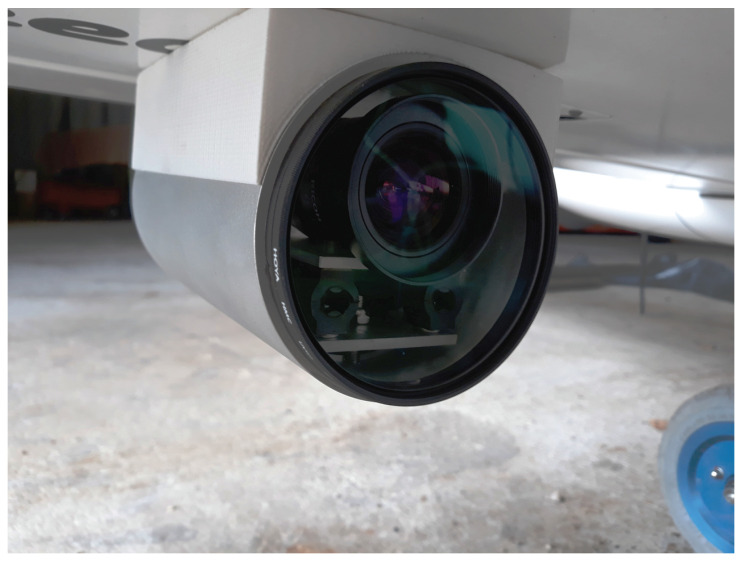
Camera under the wing of K50 aircraft. Metallic tube and replaceable filter provide physical defense while vibration dumpers reduce blur effects caused by high-frequency shakes.

The two cameras are placed under the wings, while the image processing modules reside inside the bottom payload bay of the K50 aircraft. Monocular camera sensors are placed in metallic tubes which have 3D-printed plastic holders. The cameras and optics lay on a balanced plate with vibration dumpers and an IMU unit is also fixed to the plate for supporting IMU-based calibration (Figure 9).

Intrinsic parameters of the cameras were defined through the classical checkerboard calibration method [48]. Beyond focal length and principal point, 2nd and 4th order radial distortion parameters were identified. Lenses with minimum radial distortion were used, because on-line undistortion of the image is computationally expensive. With small distortion, the line features can be detected in the original image, and only some selected feature points should be undistorted to get exact pinhole representation for homography or other navigation data calculations.

Identification of extrinsic parameters of a camera (aircraft body system $B_{NED}$ to camera $C_{XYZ}$) is a more challenging task. The simplest but less accurate way is to fix a 3rd camera near the central IMU with known relative pose, and stereo calibrations are possible to the three pairings to get 6D poses of each camera relative to the remaining two which gives the external parameters. A more precise solution is to add IMU units next to each camera, have a fixed checkerboard in FOV and move the aircraft which supports a Camera-IMU calibration [18,49]. It is interesting that even the runway marker can be used for calibration this way. We obtained 2–3 cm precision with the first method which was suitable for our navigation sensor setup which operates at 30–600 m distances, orientation was also measured by an IMU-only approach.

Image processing has an inevitable time delay which must be considered in any vision-based navigation system. The SZTAKI sensor sends two types of messages. At time of exposure it sends a timestamp message, and later the corresponding image processing results. This makes able the Kalman filter with time delay to prepare the filter for the upcoming delayed measurement. Results contain 3D position and Euler angles of the aircraft with the raw coordinates of three key features of the runway in the image (pixel coordinates of left and right corner points and the vanishing point of the side lines). These features can be derived directly from the threshold marker representation. The vision sensor was able to operate at 15 FPS, however 10 FPS was used during flight tests to provide stable operation with approximately 60 ms delay.

## 5. Real Flight Results

In this section, two real flight test data sets are discussed. The first one is the log of real-time on-board calculations made by the SZTAKI sensor during flight tests in Septfonds private airfield (France) with the VISION K50 UAV. The second data set is the imagery and state log of one approach to LOAN 09 runway (Wiener Neustadt/Ost Airport), which was provided by the C2Land Phase B team for comparison of results [11].

### 5.1. Septfonds Own Flight Tests

**Figure 10:**
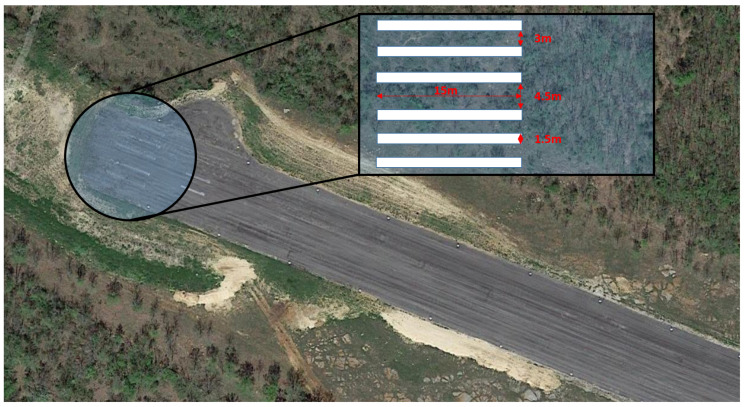
Septfonds private airfield is perfect for fixed-wing UAV prototype testing; however, its original painting was not sufficient for visual navigation. Septfonds runway with the PVC markers can be seen in Figure 2.

Flight tests of our VISION project were conducted at Septfonds private airfield in France during summer 2019 and 2020. The runway is 731 m long and 31 m wide, which is similar to a small commercial runway for manned aircraft; however, the painting of the runway was not sufficient for visual detection as can be seen in Figure 10. Old faded markers must be repainted and maintained if we want to use optical relative navigation sensors; furthermore, it is recommended to add more visual markers to aid these sensors because it is still cheaper than the installation of other guidance support systems. All-weather 0–24 solutions must have IR or UV markers, but first we need to prove the applicability and usefulness of optical navigation sensors with RGB/mono cameras before such investments. At Septfonds, white vinyl (PVC) panels were placed on the markings to mimic a well-painted runway. All the realized flight tests were manual flights; however, K50 was completely ready for autonomous flight and its large size required restricted airspace for tests which does not allow us to fly beyond line of sight, thus the approaches of the flight tests were started from 500–600 m from the threshold of the runway.

**Figure 11:**
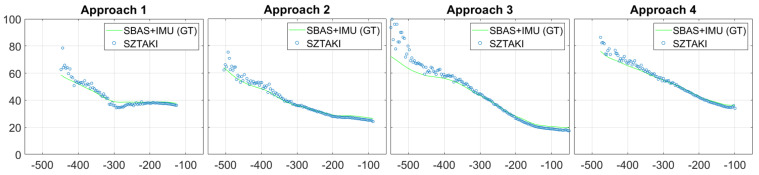
Runway relative altitude $\left(-Y_{R}\;\left[m\right]\right)$ measurements of four consecutive runway approaches at Septfonds.

Here, we discuss the results of the last test day with 4 consecutive approaches. The K50 did 5 complete circle with 5 approaches (first one was just a warm-up trial for the pilot) and there were no false detections between the approaches. Altitude measurements are shown in Figure 11, which were provided by the master sensor with the narrow 32.8 degree optics. The sensor becomes accurate at approximately 400 m. Unfortunately, these tests cannot give information about the detection range, because of the restricted test range at about 600 m. The measurements were continuous at 10 Hz and had 60 ms delay which was added by the on-board image processing.

**Figure 12:**
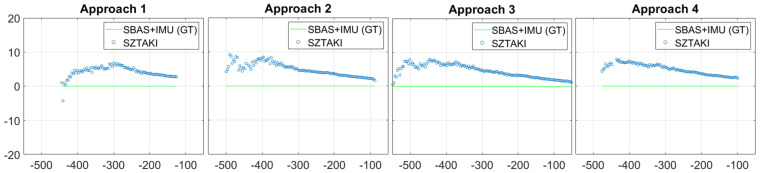
Lateral $\left(X_{R}\;\left[m\right]\right)$ error of four consecutive runway approaches at Septfonds.

We had an assumption that a precise runway marker detection gives us plenty of good corner features with known 3D coordinates on the same plane surface, thus we do not need to deal with the side lines of the runway (Septfonds runway has no side lines just white nodes). Lateral errors as presented in Figure 12 were larger than expected, and the reason maybe the 15 m threshold bars are too short (side lines would be 700 m long). The error has small deviance which also suggest that higher precision can be obtained through vision. Unfortunately, even the threshold bar area of Septfonds is not completely plane, and the carbon fiber body of the UAV may have small deformations during flight compared to on-ground calibration. The main advantage of visual navigation is that the errors are decreasing during the approach and we got 2 m lateral error at 100 m from the threshold line. Our aim was the analysis of raw optical navigation results, thus we did not use the flight data to fit our camera extrinsic parameters and runway plane parameters to the on-board GT.

**Figure 13:**
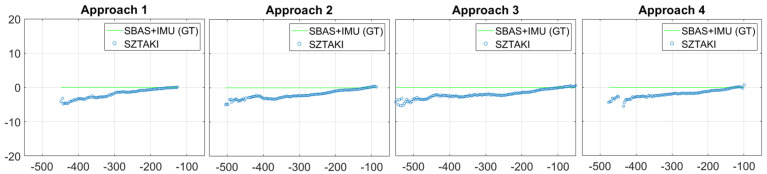
Longitudinal $\left(Z_{R}\;\left[m\right]\right)$ error of four consecutive runway approaches at Septfonds.

Longitudinal distance is the largest in absolute value among the three runway relative position coordinates, thus the same or even better performance compared to lateral errors means better relative accuracy. Figure 13 presents the longitudinal errors for the four approaches which converges to cm deviation at 100 m. The last measurement of approach 4 with increased error comes from a partial detection of the runway marker (missing of the half of the marker is allowed, and marker goes out of FOV at the end of the approach). Table 1 summarizes the bias and standard deviation of optical navigation data. The bias is distance-dependent, thus standard deviations in the table are larger than the real disturbance (bias change added); however, in an error model we need to define a bias level (mean of errors inside the interval).

**Figure 14:**
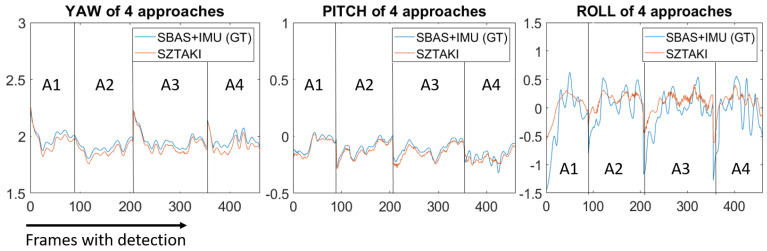
Septfonds euler angle measurements. Approaches have different duration, frames are captured at 10 Hz.

Homography of the threshold marker gives us a complete 6D relative pose of the camera from which 6D pose of the aircraft can be calculated directly. Figure 14 shows the euler angles of the aircraft during the four approaches. The yaw and pitch angles were precise with a bias, however, roll angle measurements could not follow fast oscillations. The engine of K50 has various shake frequencies (rotor rpm dependence), thus the vibration dumpers sometimes allow high-frequency shakes of the camera which can be also seen as small amplitude high frequency oscillations on the roll angle, but the large low-pass effect may also come from the dumpers, because the cameras were on the wings and roll of the UAV pushes the dumpers. Using dumpers is a trade-off because high-frequency shakes cause blur effect in the image which can prohibit detection and decrease precision of image features; however, dumpers may degrade the roll angle accuracy. In real-life experiments, the GT can also have problems, which cannot be completely excluded. Yaw bias is larger than a bias, which can come from the difference of on-ground extrinsic calibration compared to the in-flight situation. The possible other sources are the not completely planar threshold bar area, or an error in our measurement when we defined the 3D attitude of the plane of the threshold area. It does not come from the image processing, because it has no distance dependence.

Our runway detector worked precisely in the Septfonds use case real-time on-board during 600 m long approaches to an idealistic runway marker. In the next subsection, we test our image processing approach on images of a real commercial airport.

### 5.2. Wiener Neustadt/Ost C2Land TU Braunschweig Data

For validation and comparison of our runway detector, we asked the C2Land (Technical University Braunschweig) team for further camera measurements [11]. They provided us complete sensor data of one approach to the LOAN 09 runway at Wiener Neustadt. We updated the camera inner and outer parameters, and the runway marker representation (bar size and relative positions) according to their setup and target runway. The contrast of the markers was poor compared to our white PVC markers and images were given in .jpg format (compression effects), thus some parameter change was required in bar candidate detection. Higher number of bar candidates does not degrade the performance of the detector because of the fast and robust representation matching.

The aim of VISION project was the integration of a completely independent vision sensor into a safety enhanced navigation filter (just approach no landing), thus IMU data were not used in the image processing, and orientation angles were also defined by the vision sensor (6D navigation state). C2Land project created a complete vision-based navigation solution from 2000 m until landing. They use IMU orientation measurements to aid image processing, and only 3D position is calculated by the vision sensor. Threshold bar detection becomes possible from approximately 600 m thus complete runway detection is necessary from longer distances. At approximately 60 m, the threshold bars go out of the camera image and only center line and side lines are visible, which can only define vertical and lateral displacement. Here, we give the comparison of the C2Land RGB results (4-point runway model match) to the SZTAKI RGB image processing (threshold marker representation match) in the case of visible threshold markers (600 m to 60 m from runway threshold).

It is sure that features with bigger physical size can be detected from longer distances, and a complex marker can be detected more accurately and robustly without an initial guess if it can be seen. The first detection of the threshold marker by SZTAKI sensor is at 718 m but the detection become fully continuous from 434 m.

**Figure 15:**
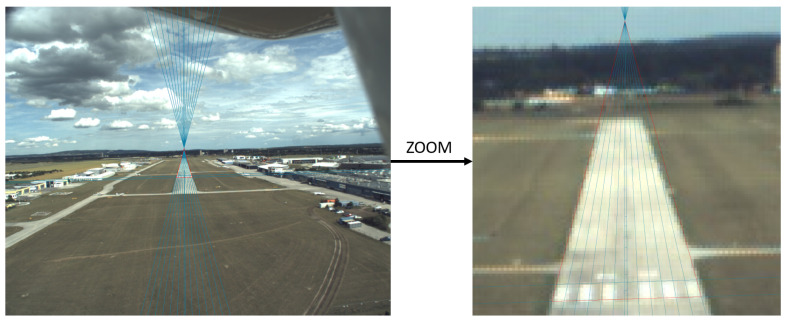
Image taken 434 m from runway threshold. The edges of bar markers are uncertain compared to the runway edges.

Figure 15 shows the image which was taken from 434 m with the result of the threshold marker detection. For this particular image, a 4-point runway model would provide more accurate solution because the bars are short and have uncertain edges which leads inferior complete fit. We can assume that this marker detection can give a reasonable initial guess to C2Land runway fit, thus we can also derive a combined IMU independent method for longer distances (for 400–600 m).

**Figure 16:**
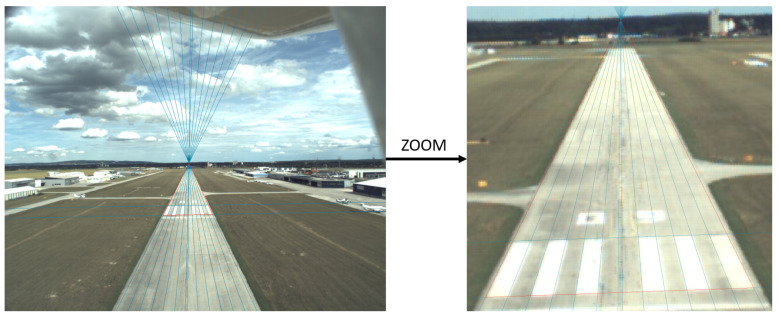
Image taken 100 m from runway threshold. The bar markers perfectly detectable. Runways are not completely flat which can cause inaccuracy in the case of complete runway detection from closer positions <200 m.

Figure 16 shows a case where threshold marker is well visible at 100 m. The homography of the bars gives an accurate and stable solution for the relative position and orientation of the camera. The assumption of a flat runway is more likely true for the threshold bar area compared to the whole structure which has often a bump at the middle. Our main question here is to find out when one should use the complete runway model and when the threshold marker representation.

**Figure 17:**
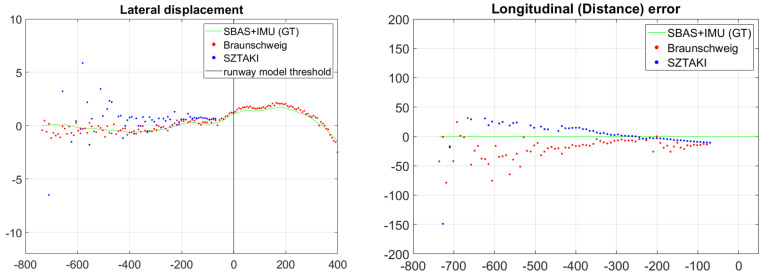
Lateral displacement according to distance from runway and the error of distance measurements. Threshold markers are no longer visible within 60 m because the camera looks ahead of the markers, however, lateral displacement can be calculated with center line and side line detection.

Measurement accuracy is presented for lateral and longitudinal relative distance in Figure 17. The SZTAKI sensor has superior longitudinal accuracy within 600 m; however, it is inferior in lateral accuracy even from short distances (lateral error is much better than at Septfonds). Lateral errors are very low for both sensors within 400 m, and the SZTAKI sensor error may come from additional small orientation errors (C2land has IMU orientation). C2Land lateral results clearly better above 400 m longitudinal distance which comes from the uncertainty of edges of short bars in images from >400 m distances.

**Figure 18:**
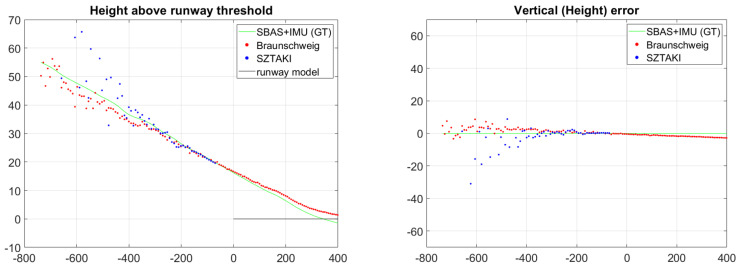
Altitude is the most important navigation data for the aircraft. Braunschweig measurements start at 2000 m with IR-based detections. The SZTAKI sensor first detection is at 718 m and become fully continuous from 434 m. The sign of vertical error is defined by the downward looking $Y_{R}$ axis of the runway frame.

Altitude results are the most critical for safety because altitude is in close connection with height above the ground. Figure 18 presents the altitude measurement results and their errors. According to this comparison, we can conclude that threshold marker representation becomes comparable to runway model fit at 400 m and superior at 200 m with 63.35 degree optics, 1280 × 1024 image resolution, and 23 m runway width. The IMU-free method can be used within 600 m with threshold bar detection as initial guess for complete runway model fit. These results also demonstrate that vision-based navigation raw results can outperform GPS altitude precision (within 200 m) at the most critical phase of the approach. Table 2 shows the detailed comparison of SZTAKI and Braunschweig navigation results. GPS alone has meter range altitude errors while SBAS improves it to around 30 cm altitude errors, thus optical sensors are extremely useful at the end of the approach. Within 60 m longitudinal distance when the threshold marker is no longer visible, only the side lines and center line can be used for visual navigation which can provide only lateral and vertical measurements. C2Land vision-based vertical measurements indicate good performance within 50 m from threshold and during landing.

### 5.3. Comparison of 32.8 and 60.97 Degree Optics

Narrow FOV optics give better angular resolution with the same camera sensor, thus we get more accurate navigation results from the image features. Furthermore, narrow FOV makes the detection of the same physical feature possible from longer distances. The main disadvantage of narrow FOV camera is the increased dependence on possible trajectories. Large deviation from ideal trajectory can push the image of the runway out from the FOV. This is a crucial problem, because we would like to increase robustness of the navigation system, and with a too narrow optics we have no vision-based information in the most important situations when something goes wrong with the trajectory. This comparison of navigation results with a 32.8 and 60.97 degree optics on the same UAV can give some insight into the real trade-off.

**Figure 19:**
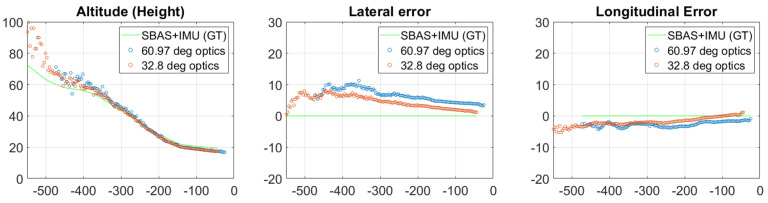
Measurements of relative positions with different optics during approach 3 of the Septfonds flight test.

Figure 19 shows the relative position measurements of one approach of VISION K50 to Septonds runway. Detection of a 31 m width runway starts at 470 m with the 60.97 degree optics, while 32.8 degree optics can detect runway markers from 550 m. The wide FOV optics can see the threshold bars 20 m further at the end of the approach and has comparable accuracy from 200 m. From longer distances (>400 m), 32.8 degrees is absolutely beneficial for threshold bar detection; however, even small orientation deviance can lead to the out of FOV problem. Nearby the runway, where the most precise solution is required, the 60.97 degree sensor can operate longer and provides the necessary precision for altitude. Results on C2Land data suggest that complete runway fit is necessary above 400 m distance from threshold and lateral displacement should be calculated based on side line detection near the runway, thus 60 degree FOV seems to be a better choice.

## 6. Conclusions

This paper introduces an optical navigation sensor for fixed-wing aircraft during final approach to a runway with a visible threshold marker. Vision-based navigation sensors are additional building blocks for INS/GNSS sensor fusion. Raw precision of vision sensors is still an important issue. Robust and continuous detection of threshold marker is solved by representation matching of quadrangle candidates coming from a Sobel-based edge chain pairing. After threshold marker initial detection, homography can generate a top-view projection of the threshold marker area on which fine-tuning of key corner markers is possible. A complete hardware solution was also designed for real flight tests. On-board real-time performance was 10 Hz stable (15 Hz free run avg.) full frame (2048 × 1536) detection with 60 ms image processing delay.

Navigation data of the vision sensor were compared to the GNSS(SBAS)/INS sensor fusion results, which confirmed high precision for longitudinal distance and height within 400 m and acceptable precision for lateral displacement (lateral error is also precise for C2Land data within 400 m). Yaw and pitch angles are also calculated precisely, however, roll angle measurements were degraded by the vibration dumpers. Image processing approach was also compared to the competitive C2Land project results, which confirmed that threshold marker based approach is beneficial within 400 m, however, side line detection can enhance lateral accuracy. The threshold marker detector can also give good estimates within 600 m for the ROI-based complete runway fitting methods.

One of the most important design parameters is the FOV of the optics. Comparison of 32.8 degree and 60.97 degree optics suggests that using a larger FOV is better for operational robustness while narrow optics gives mainly additional detection range. Accuracy improvement is significant only for long distance measurements. Large idealistic (plane surface, no dirt) visual markers can enhance the robustness and precision of runway relative navigation; the methods which are described in this paper can be applied for other patterns, for instance, threshold bars with touch down markers or other fixed visual features nearby the runway.

## Figures and Tables

**Table 1:** Bias and standard deviation of navigation data in different distance intervals at Septfonds (average of 4 approaches).

Distance from Threshold	50–100 m	100–200 m	200–300 m	300–400 m	400–500 m
**altitude bias [m]**	−1.8840	−1.3204	−0.6437	1.7595	4.8201
**alt. error std. dev. [m]**	0.3062	0.2922	0.7932	1.5418	3.8109
**lateral bias [m]**	1.7876	2.8946	4.4189	5.9679	5.4848
**lat. error std. dev. [m]**	0.2512	0.3595	0.6298	0.7478	1.5177
**longitudinal bias [m]**	0.3473	−0.4825	−1.6671	−2.6347	−3.4408
**long error std. dev. [m]**	0.2445	0.3677	0.2994	0.3217	0.6386

**Table 2:** Bias and standard deviation of navigation data in different distance intervals at Wiener Neustadt/Ost (B: Braunschweig SZ: SZTAKI).

Distance from Threshold	50–100 m		100–200 m		200–300 m		300–400 m		400–500 m	
**sensor**	**B**	**SZ**	**B**	**SZ**	**B**	**SZ**	**B**	**SZ**	**B**	**SZ**
**altitude bias [m]**	−0.0737	0.2670	0.3874	0.1074	1.0896	0.2597	1.9344	−0.6320	2.5521	−2.4199
**alt error std. dev. [m]**	0.3024	0.0412	0.4698	0.2846	0.4434	0.6396	0.6664	1.7852	0.7851	6.0318
**lateral bias [m]**	0.2001	0.4220	0.1564	0.5076	0.0806	0.7510	0.0525	0.8705	−0.0174	1.4082
**lat error srd. dev. [m]**	0.2465	0.0479	0.1546	0.1174	0.2107	0.3526	0.1918	0.5429	0.3813	1.1161
**longitudinal bias [m]**	−13.6587	−9.9333	−16.6296	−6.3390	−6.5999	0.9311	−11.8413	8.3864	−20.9093	13.8631
**long error std. dev. [m]**	0.8324	0.5453	4.3249	1.8621	1.3642	1.3985	3.8408	4.4991	6.1194	2.6217

## Data Availability

The data presented in this study are available on request from the corresponding author.
